# Contribution of One‐Electron Oxidation of Purine and Pyrimidine Bases to the Photo‐ and Radiation‐Induced Damage to Cellular DNA

**DOI:** 10.1002/cphc.70457

**Published:** 2026-08-04

**Authors:** Jean Cadet, Dimitar Angelov, Paolo DI Mascio, J. Richard Wagner

**Affiliations:** ^1^ Département des sciences de l’imagerie médicale et des radiations Faculté de médecine et des sciences de la santé Université de Sherbrooke Sherbrooke Canada; ^2^ Ecole Normale Supérieure de Lyon CNRS Laboratoire de Biologie et de Modélisation de la Cellule LMBC Université de Lyon Lyon France; ^3^ Izmir Biomedicine and Genome Center Izmir Turkey; ^4^ Department of Biochemistry Institute of Chemistry Universidade de São Paulo São Paulo Brazil

**Keywords:** DNA oxidation biomarkers, DNA‐protein cross‐links, interstrand DNA cross‐links, intrastrand DNA cross‐links, oxidized nucleobases, radical cation

## Abstract

One‐electron oxidants that include type I photosensitizers, biologically relevant carbonate anion radical generators, and high intensity UVC nanosecond lasers are able to ionize in aqueous solution purine and pyrimidine nucleobases. The survey is aimed at critically reviewing the available information on the fate of initially generated base radical cations that essentially involve deprotonation and nucleophilic addition reactions. Accumulated evidence leads to the conclusion that one‐electron oxidation reactions of nucleobases partly mimic the oxidizing features of hydroxyl radical, the main biologically relevant oxidant, in terms of formed degradation products. In contrast to the relatively nonspecific attack of hydroxyl radicals, guanine sites are the main targets of one‐electron oxidation. This may be rationalized by efficient redistribution of base radical cations through charge transfer along double‐stranded DNA with preferential final product formation at guanines, as illustrated by the overwhelming formation of 8‐oxo‐7,8‐dihydroguanine. Interestingly, a similar observation was made for cellular DNA; thus, providing strong support for the occurrence of hole migration in model DNA substrates as well as in cells. The specific distribution of DNA degradation products displayed by one‐electron oxidants was used to provide further support for the modest role of direct effects of ionizing radiation in the oxidative degradation of cellular DNA.

## Graphical abstract

One‐electron oxidation of purine and pyrimidine bases of isolated and cellular DNA generates related reactive base radical cations that are converted into final decomposition products through deprotonation and hydration. In the case of guanine, the most susceptible nucleobase to one‐electron oxidants, 8‐hydroxy‐7,8‐dihydroguanyl radical, thus formed by hydration of Gua**·** ^+^, gives rise to predominant 8‐oxo‐7,8‐dihydroguanine (8‐oxoGua) and 2,6‐diamino‐4‐hydroxy‐5‐formamidopyrimidine (FapyGua) by oxidation and reduction, respectively.

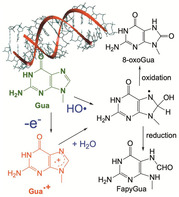

## 1 Introduction

Endogenous oxidation of biomolecules, including DNA, is a ubiquitous process in living cells under physiological conditions. Thus, cellular oxidative metabolism triggered by mitochondrial respiration [1, 2, 3] is one of the main biological sources of superoxide anion radical (O_2_ **·** ^−^)/hydroperoxyl radical (HO_2_ **·**), both poorly reactive oxygen species (ROS) [3, 4, 5] that are converted into hydrogen peroxide (H_2_O_2_), another ROS by spontaneous and enzymatic dismutation. However, the reduction of H_2_O_2_ by transition metals such as ferrous ion according to the so‐called Fenton reaction gives rise to highly reactive hydroxyl radicals (**·**OH, E^0^ = 2.7 V vs NHE) [6, 7]. It should be noted that the formation of **·**OH and its precursors is exacerbated under various processes, including aging, phagocytosis, and diverse pathological situations, such as diabetes, cancer development, neurodegeneration, and cardiovascular disease [8, 9, 10, 11]. Ionizing radiation is another major source of **·**OH in aqueous environments/cellular medium through ionization of water molecules [12]. Interestingly, **·**OH efficiently reacts at the site of its formation with the nearest molecules at diffusion‐controlled rates of reaction. Abundant literature is available on the main **·**OH‐mediated degradation pathways of nucleobases [13, 14] and the 2‐deoxyribose moiety [15, 16] of DNA in aerated aqueous solutions as the result of initial hydrogen atom abstraction from C—H bonds and addition to C=C and C=N bonds. Two other ROS are able to oxidize DNA bases in more selective ways: 1) singlet oxygen (^1^O_2_), that is formed by type II photosensitizers through triplet energy transfer to molecular oxygen, which exclusively reacts with guanine (Gua) to specifically generate 8‐oxo‐7,8‐dihydroguanine (8‐oxoGua) [17]; and 2) peroxyl radicals that are produced from the addition of oxygen to intermediate pyrimidinyl and 2‐deoxyribos‐5‐yl C‐centered radicals, which preferentially add to 5′‐flanking 2′‐deoxyguanosine (dG) in oligodeoxyribonucleotides/DNA giving rise to tandem modifications [18]. Other specific types of oxidative biological reactions involve TET enzymes that iteratively oxidize 5‐methylcytosine (5‐mCyt) into 5‐hydroxymethylcytosine (5‐HmCyt), 5‐formylcytosine (5‐foCyt), and 5‐carboxycytosine [19, 20, 21, 22] (5‐CaCyt) as part of an active demethylation pathway, particularly in embryonic stem cells and neurons. In addition, major efforts have been devoted to unraveling the oxidative mechanisms of DNA degradation upon exposure to chemical and physical one‐electron oxidants in aerated aqueous solutions. The aim of this short review is to critically survey mechanistic aspects of the fate of initially generated pyrimidine and purine radical cations of model compounds consisting mainly of 2′‐deoxribonucleosides and to a lesser extent of dinucleoside monophosphates. This was mostly achieved through the characterization of the final degradation products used as biomarkers for their search in both isolated and cellular DNA. The information thus gained on the distribution of base oxidation products in genomic DNA has been used to estimate the contribution of direct and quasi‐direct effects of ionizing radiation on nucleobases with respect to the indirect effect of **·**OH generated by the radiolysis of water molecules.

## 2 One‐Electron Oxidation of the Nucleobase Moieties

Purine and pyrimidine nucleobases are the selective targets of most one‐electron oxidants with a susceptibility that decreases in the following order (G > A > T ∼ C), which is inversely correlated with the reduction potential values versus NHE of related 2′‐deoxyribonucleosides: dG (E = 1.29), dA (1.42), dT (1.7) dC (1.6) [23, 24]. One major exception concerns ionizing radiation that is able to abstract an electron not only from nucleobases [12, 25] but also from the 2‐deoxyribose moiety of DNA [15]; thus, triggering the formation of oligonucleotide single strand breaks (SSBs) and oxidized abasic sites, including 2‐deoxyribonolactone and C4‐hydroxylated abasic sites [16].

### 2.1 Chemical and Physical One‐Electron Oxidants

‐ Far UV photolysis. Evidence was provided in the early 1990's that 193‐nm laser photolysis is able to trigger the ionization of guanine model compounds [26]. It was shown a few years later that Fpg‐sensitive sites were generated as guanine modifications upon exposing isolated DNA to 193‐nm light [27]. UVC irradiation is a poor ionizing agent of essentially guanine bases [28, 29] with, however, a reported higher efficiency for human telomeric G‐quadruplexes [30]. 8‐OxoGua, a predominant one‐oxidation product of guanine, is formed in very low yields upon UVC irradiation of isolated dG [29] and cellular DNA [31].

‐ Type I photosensitizers. Early studies on one‐electron oxidation of nucleobase components have involved type I photosensitizers (for a review see et al. Baptista [32]) including among others 2‐methyl‐1,4‐naphthoquinone (MQ) [33, 34], riboflavin [35], benzophenone [36] and transition metal complexes [37, 38] that in their triplet excited state are able to abstract an electron from nucleobases. With the exception of excited MQ [39, 40], guanine, which exhibits the lowest oxidation potential among DNA bases [23], is the preferential target of isolated nucleosides. Anthraquinone and MQ tethered to the 5′‐end of oligonucleotides have been used as suitable photosensitized one‐electron oxidants of the proximal GG step for the injection of a positive hole in DNA duplexes [41] to investigate hole transfer reactions within DNA duplexes [40, 42, 43].

‐ Photochemical generation of sulfate radical anion (SO_4_ **·** ^−^, E^0^ = 2.43 V vs NHE) and carbonate radical anion (CO_3_ **·** ^−^, E^0^ = 1.59 V vs NHE) [44, 45, 46]. SO_4_ **·** ^−^, that is formed by nanosecond (ns) 308‐nm laser irradiation of aqueous solutions of peroxydisulfate anions (S_2_O_8_^2−^) is able to oxidize pyrimidine and purine bases [47]. The presence of an excess of sodium bicarbonate in S_2_O_8_^2−^ solutions allows for the quantitative formation of CO_3_ **·** ^−^ as a selective one‐electron oxidant of guanine [45]. Both methods are suitable for performing one‐hit oxidation experiments upon controlled exposure of nucleosides/DNA to a single laser pulse.

‐ Biological source of CO_3_ **·** ^−^. Early findings have shown that poorly reactive O_2_ **·** ^−^ and nitric oxide (**·**NO), enzymatically generated in cells under oxidative stress conditions, including inflammation and aging, efficiently combine (k ∼ 10^10^ M^−1^ s^−1^) [48] to form peroxynitrite (ONOO‐) in equilibrium with its protonated form, a potent oxidant [49, 50]. In the presence of physiological amounts of CO_2_/bicarbonate that efficiently react with ONOO‐/ONOOH, instable nitrosoperoxycarbonate (ONOOCO_2_^−^) is formed [51, 52] that upon fast homolysis [53, 54] gives rise to CO_3_ **·** ^−^ and nitric dioxide (**·**NO_2_) that is able to oxidize and nitrate guanine [55], tryptophan and tyrosine [56] among biological targets.

‐ Bi‐photonic ionization of pyrimidine bases. High‐intensity ns and pico‐second 266‐nm laser irradiation has been shown to be a relevant way for generating nucleobase radical cations through a bi‐photonic ionization process (for early reviews, see Nikogosian [57] and Angelov et al. [58]). More recently, evidence was provided showing that the ionization of purine and pyrimidine nucleobases occurs with a similar efficiency [59].

‐ Sodium bromate (NaBrO_3_). Reduction of the food additive NaBrO_3_, a rodent renal carcinogen [60] by thiols triggers the formation of NaBrO_2_, which is a selective one‐electron oxidizing agent toward guanine in DNA [61, 62, 63]. NaBrO_3_, which is widely used to generate 8‐oxoGua in cellular DNA [64, 65, 66, 67], has recently been suggested as a suitable oxidant for the calibration of the formamidopyrimidine DNA *N*‐glycosylase (Fpg)‐modified comet assay [68]. In addition to 8‐oxoGua, other oxidatively generated lesions of guanine have also been reported to be formed upon exposure of DNA to one‐electron oxidants, including 2,6‐diamino‐4‐hydroxy‐5‐formamidopyrimidne (FapyGua), 2,2,4‐triamino‐5‐(2*H*)‐oxazolone (Oz), and DNA‐protein cross‐links (DPCs) [14]. The lack of consideration of the latter modifications is likely to affect the suitability of previous conclusions on the repair and mutagenic features of 8‐oxoGua when BrO_3_ is used as the oxidant.

### 2.2 Base Oxidation Modifications (Model Compounds)

Most type I photosensitization mechanistic studies of nucleobases have involved 2′‐deoxyribonucleosides and short DNA fragments. The use of 2′‐deoxyribonucleosides instead of nucleobases presents several advantages that are listed below:

‐ Higher solubility of 2′‐deoxyguanosine (dG) in aqueous solution.

‐ Ability to separate the individual oxidation products of 2′‐deoxyribonucleosides by reversed phase chromatography; in comparison, it is not possible to separate with baseline resolution oligonucleotides containing various oxidation products that are longer than 3–4 nucleotides

‐ The presence of a 2‐deoxyribose substituent at N1 of thymidine (dT) prevents the preferential formation of thymin‐1‐yl radical [69]. If not, the overwhelming radical reacts effectively with thymine in aerated aqueous solutions to generate isomeric adducts [34, 70].

‐ Higher susceptibility of 2′‐deoxyribonucleosides to electrospray ionization (EI) and fragmentation that improves the detection of oxidized nucleosides by HPLC‐tandem mass spectrometry analysis of oxidized cellular DNA.

‐ Isolated dG is not an appropriate model compound for one‐electron oxidation studies since 8‐oxo‐7,8‐dihydro‐2′‐deoxyguanosine (8‐oxodG), one of the main initially DNA‐formed oxidation products, is continuously consumed as soon as it is generated by the highly oxidizing neutral guanine radical (G‐H)**·** [71] arising from the fast deprotonation of the guanine radical cation (G**·** ^+^) [72].

#### 2.2.1 Main Reactions of Pyrimidine and Purine Radical Cations

Nucleobase radical cations are highly reactive intermediates that rapidly decay in aqueous solutions according to two main competitive reactions consisting of hydration and deprotonation with rates that strongly depend on the single‐ or double‐stranded state of DNA.

Deprotonation of the base radical cation of dT and 5‐mdC that predominantly involves the exocyclic methyl group gives rise, respectively, to 5‐(uracilyl)methyl and 5‐(cytosinyl)methyl radicals [34, 73, 74, 75] as the precursors of related hydroperoxyl radicals in aerated aqueous solutions. One may note that the base radical cation of dC deprotonates at the 4‐amino group of the base as well as at C1 of the 2‐deoxyribose moiety to generate 2′‐deoxyuridine (dU) and 2‐deoxyribonolactone, respectively [76]. Time‐resolved spectroscopic measurements have shown that deprotonation of purine base radical cations is a fast event [25, 77] in aqueous solution. Thus, G**·** ^+^, a Bronsted acid (p*K*_a_ = 3.9), predominantly deprotonates in isolated dG to give (G‐H)**·** with a rate (k_dp_ = 1.8 × 10^7^ s^−1^) at neutral pH [78]. In comparison, the rate of proton loss is slower in ds‐DNA, and as well the rate varies tenfold depending on sequence (*k* = 10^6^ ∼ 10^7^ s^−1^) [79]. The released neutral aminyl radical G(N1−H)**·** [25] upon tautomerization is in dynamic equilibrium with guanine transients, including a C5‐carbon‐centered radical. The latter radical exhibits poor reactivity toward O_2_ (k ∼ 10^2^ M^−1^ s^−1^) [80] yet reacts efficiently with O_2_ **·** ^−^ (*k* = 3 = 10^9^ M^−1^ s^−1^) [81, 82]. Such reactivity is similar to other highly oxidizing biochemical radicals that are produced by one‐electron and **·**OH oxidation of tryptophan and tyrosine [83, 84]. Deprotonation of the adenine radical cation [26, 85, 86, 87] occurs at the exocyclic 6‐amino group, giving rise to a long‐lived aminyl radical (A‐H6)**·** that, as (G‐H)**·**, does not react with O_2_.

Hydration of the pyrimidine radical cation of dT occurs predominantly at C6 to produce 6‐hydroxy‐5,6‐dihydrothymin‐5‐yl [34, 73], a minor intermediate of **·**OH addition across the 5,6‐double bond. A similar reaction is noted for dC [73, 75], with however, a lower specificity for addition at C6, explaining the formation in low yields of 6‐hydroxy‐5,6‐dihydrocytos‐5‐yl radical. Nucleophilic addition of H_2_O that occurs selectively at C8 of the imidazole ring of both adenine and guanine radical cations leads to the formation of reducing 8‐hydroxy‐7,8‐dihydropurin‐7‐yl radicals [72, 88, 89]. Importantly, hydration of G**·** ^+^ in isolated nucleoside/nucleotide occurs with an estimated rate of 5 orders of magnitude lower than deprotonation [90]. However, competitive hydration of G**·** ^+^ is also significant in duplex DNA with a sevenfold increase with respect to ss‐oligonucleotides [91], G‐quadruplex basket and hybrid conformations [92]. This is partly explained by the fast occurrence of a proton transfer equilibrium with cytosine as the base pair on the opposite strand and a favored exposure of the C8 carbon to water molecules.

As discussed above, decay of initially generated base radical cations gives rise to neutral radicals through competitive water addition and deprotonation reactions. It should be noted, however, that the resulting nucleoside/nucleotide transient radicals exhibit major differences in their reactivity toward O_2_. Thus, the neutral pyrimidine radicals, essentially C‐centered radicals, undergo efficient O_2_ addition at a nearly diffusion‐controlled rate (k ∼ 2 × 10^9^ M^−1^ s^−1^ [15, 93] to generate related peroxyl radicals. This reaction is part of the so‐called “oxygen fixation hypothesis” that has been proposed to explain the ability of O_2_ to sensitize the biological effects of ionizing radiation [94]. In contrast, peroxyl radicals are not formed during the decay of adenine and guanine radical cations in aerated aqueous solutions due to the lack of O_2_ addition to intermediate neutral purine iminyl and aminyl radicals. Nevertheless, O_2_ is able to one‐electron oxidize 8‐hydroxy‐7,8‐dihydroguan‐7‐yl radicals (*k* = 4 × 10^9^ M^−1^ s^−1^), leading to the formation of 8‐oxo‐7,8‐dihydroguanine. As a competitive reaction that is favored in the absence of O_2_, the latter radical undergoes one‐electron reduction, leading to rapid opening of the imidazole ring (*k* = 2 × 10^5^ s^−1^) and the formation of 2,6‐diamino‐4‐hydroxy‐5‐formamidopyrimidine (FapyGua [81]. Similar oxidation and reduction reactions take place for 8‐hydroxy‐7,8‐dihydroaden‐7‐yl radical in aerated aqueous solutions.

#### 2.2.2 Thymine

**FIGURE 1:**
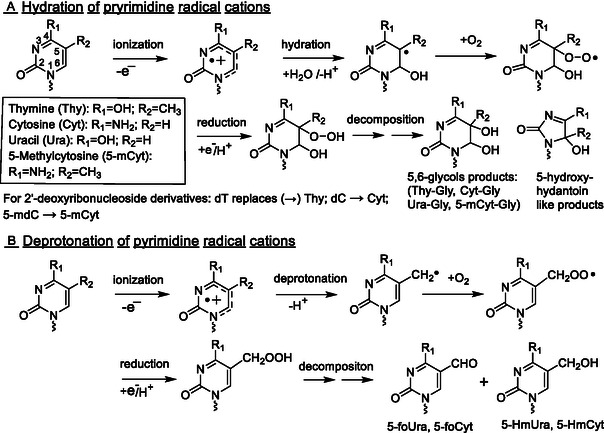
One‐electron oxidation of pyrimidines. Top panel A shows the hydration of pyrimidine radical cations for thymine, cytosine and 5‐methylcytosine in DNA. As shown, hydration involves the addition of water at C6 of the pryrimidine 5,6‐double bond to give 5,6‐glycol products and 5‐hydroxyhydantoin like products. The 5,6‐glycols of Cyt (Cyt‐Gly) are not stable and undergo dehydration into 5‐hydroxycytosine (5‐OH‐Cyt) and deamination into the 5,6‐glycols of uracil (Ura‐Gly) suppressed (not shown). Similarly, 5‐mCyt‐Gl undergoes deamination into Thy‐Gly. Bottom panel B shows illustrates the main competitive pathway of hydration, which involves deprotonation of pyrimidine radical cations. Deprotonation occurs from the methyl group of Thy and 5‐mCyt leading to the corresponding neutral pyrimidinyl methyl radical species, which in turn gives methyl oxidation products in the presence of oxygen (5‐foUra, 5‐HmUra, 5‐foUra, 5‐foCyt). In contrast, Cyt radical cations undergo deprotonation at either the exocyclic amino group or at C1’ of the 2‐deoxyribose moiety to give uracil or N–glycosidic bond cleavage with 2‐ribonolactone, respectively. Several structures are abbreviated in the text as the corresponding 2’‐deoxyribonucleoside: including the 5,6‐glycol products of thymidine (dT‐Gly), 2’‐deoxycytidine (dC‐Gly) and 5‐methy‐2’‐deoxycytidine (5mdC‐Gly). The same applies for 5‐foUra (5‐fodU), 5‐foCyt (5‐fodC), 5‐HmUra (5‐HmdU) and 5‐HmCyt (5‐HmdC).

The main degradation products of the one‐electron oxidation of the base moiety of thymidine (dT) have been isolated and extensively characterized (see Figure 1 for structures and proposed pathways of pyrimidine radical cations) [95]. Five hydroperoxides consisting of a pair of *cis* and *trans* diastereomers of 5‐hydroperoxy‐6‐hydroxy‐5,6‐dihydrothymidine and the stable 5‐(hydroperoxymethyl)‐2′‐deoxyuridine (5‐HpmdU) are the main primary oxidation products formed by MQ‐UVA sensitization of dT in aerated aqueous solutions [96]. In addition, several stable oxidation products that result from the decay of transient 5‐hydroperoxyl radical and subsequent decomposition of the corresponding hydroperoxides have been identified, including the 4 *cis* and *trans* diastereomers of 5,6‐dihydroxy‐5,6‐dihydrothymidine (dTGly), 5‐(hydroxymethyl)‐2′‐deoxyuridine (5‐HmdU), and 5‐formyl‐2′‐deoxyuridine (5‐fodU) [95]. In addition, the nucleoside derivatives of 5‐hydroxy‐5‐methylhydantoin and *N*‐formamide are formed through cleavage of the 5,6‐bond of unstable 5,6‐hydroxyhydroperoxides, followed by rearrangement and partial hydrolysis of the intermediate ureids, respectively.

#### 2.2.3 Cytosine/5‐Methylcytosine

The main one‐electron oxidized products of the base moiety of dC arise from the decay of predominantly generated 6‐hydroxy‐5,6‐dihydrocytos‐5‐yl radicals (Figure 1). The main products consist of 5‐hydroxy‐2′‐deoxycytidine (5‐ohdC) and 5,6‐dihydroxy‐5,6‐dihydro‐2′‐deoxyuridine (dU‐Gly) [97] that result from the respective deamination and dehydration of *cis* and *trans* 5,6‐dihydroxy‐5,6‐dihydro‐2′‐deoxycytidine (dC‐Gly) [98], the reduction product of unstable 5‐hydroperoxy‐6‐hydroxy‐5,6‐dihydro‐2′‐deoxycytidine [75, 97]. In addition, the latter 5‐hydroperoxide decomposes into *N*1‐(2‐deoxy‐*β*‐D‐*erythro*‐pentofuranosyl)‐5‐hydroxyhydantoin and *N*‐formamide by cleavage of the pyrimidine ring, similar to the 5,6‐hydroxyhydroperoxides of dT [96]. On the other hand, a different distribution profile is associated with the generation of minor 5‐hydroxy‐5,6‐dihydrocytos‐6‐yl radicals that, upon addition of O_2_, are likely converted into labile *cis* and *trans* diastereomers of 6‐hydroperoxy‐5‐hydroxy‐5,6‐dihydro‐2′‐deoxycytidine. In this case, the 6‐hydroperoxide derivatives of dC undergo 6,4‐intramolecular cyclization into an intermediate endoperoxide, which transforms into four interconverting *cis* and *trans* diastereomers of *N*1‐(2‐deoxy‐*β*‐D‐*erythro*‐pentofuranosyl)‐1‐carbamoyl‐3,4‐dihydroxy‐2‐oxoimidazolidine suppressed [99]. In addition, deprotonation of dC radical cations at the exocyclic 4‐amino group and C1’ osidic moiety of free 2′‐deoxyribonucleoside leads respectively to the formation of substantial amounts of dU and to a lesser extent of 2‐deoxyribonolactone, accompanied by the release of cytosine [75, 97]. Further insights into the mechanism of deamination of the exocyclic 4‐aminyl radical were provided by a theoretical study [100]. As well, other competitive reactions of the latter radical have been identified in short DNA fragments. For example, MQ mediated UVA‐sensitization of d(CpC) generated a biadduct through the covalent binding of 4‐aminyl radical to 5‐vicinal dC at C6 [101]. It was recently shown that independently generated 2′‐deoxycytidin‐*N*4‐yl radical in a GTC sequence of double‐stranded oligonucleotides is able to abstract a hydrogen atom from the methyl group of vicinal dT with concomitant regeneration of dC [87]. This is followed by the formation of 5‐(uracilyl)methyl peroxyl radical that adds to adjacent 5′‐dG to generate a traceless tandem base lesion consisting of 8‐oxodG and 5‐fodU as previously characterized in a trinucleotide model system [102]. Evidence was provided that 2′‐deoxycytidin‐*N*4‐yl radicals are able to oxidize guanine by one‐electron in either isolated dG or in opposite GGG sequences in ds‐oligonucleotides [87].

The MQ photosensitized oxidation of 5‐mdC in aerated aqueous solutions predominantly involves the generation of the corresponding base radical cation followed by deprotonation. This is supported by the formation of three methyl oxidation products (∼60%), which were extensively characterized by MS and NMR analysis [103]. Primarily formed 5‐(hydroperoxymethyl)‐2′‐deoxycytidine (HpmdC) decays to 5‐formyl‐2′‐deoxyuridine (5‐fodC) and 5‐(hydroxymethyl‐2′‐deoxycytidine (5‐HmdC) in D_2_O solution at 293°K with a rate constant (*k* = 7.3 × 10^−2^ h^−1^) and a half‐life of 9.5 h, which is two orders of magnitude lower than that of stable 5‐HpmdU [96]. UVA‐excited MQ‐tethered oligodeoxynucleotides promoted the formation of 5‐fodC that was specifically detected at 5‐mdC sites as alkali‐labeled lesions [74, 104]. The main expected products of the hydration pathway included the *cis* and *trans* diastereomers of 5,6‐dihydroxy‐5,6‐dihydro‐5‐methyl‐2′‐deoxycytidine (5‐mdC‐Gly) that undergo a slow deamination process (*k* = 140 × 10^−4^ h^−1^) at 25°C, leading to dT‐Gly according to pseudo first‐order‐kinetics [105].

#### 2.2.4 Guanine

**FIGURE 2:**
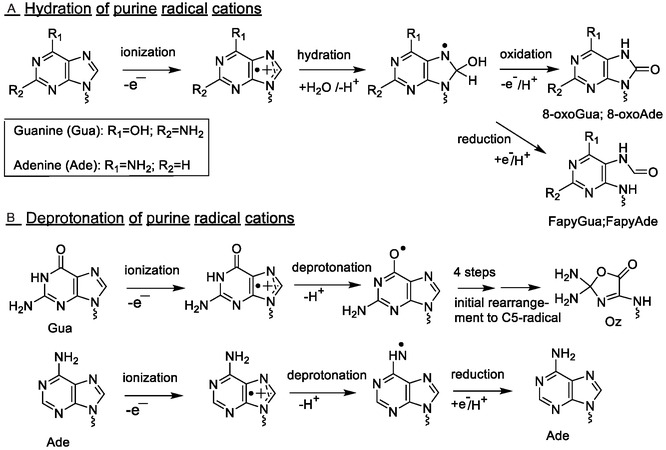
One‐electron oxidation of purines. Top panel A shows the hydration of purine radical cations for guanine and adenine in DNA. As shown, hydration takes place at C8 of the purine 7,8‐double bond leading to a neutral ambivalent radical species, which undergoes either oxidation to 8‐oxo purine derivatives (8‐oxoGua and 8‐oxoAde) or reduction to formamidopyrimidine derivatives (FapyGua and FapyAde) depending on the conditions. Bottom panel B illustrates the deprotonation pathway of purine radical cations, which is in competition with the hydration pathway. The neutral purine radical resulting from the deprotonation of Gua and Ade radical cations have different fates. In the case of Gua(‐H)^•^, the main reaction involves coupling of O_2_ **·** ^−^ with a resonant radical form at C5 of G(‐H)^•^ followed by additional steps to give 2,2,2‐triamino‐5(2*H*)‐oxazolone (Oz). The 8‐oxopurine derivatives are also abbreviated as the corresponding 2’‐deoxyribonucleoside: 8‐oxoGua (8‐oxo‐dG) and 8‐oxoAde (8‐oxodG).

Formation of 8‐hydroxy‐7,8‐dihydroguan‐8‐yl radical according to specific hydration of G^•+^ is followed by predominant one‐electron oxidation leading to 8‐oxoGua (see Figure 2 for structures and proposed pathways of purine radical cations) [106, 107, 108]; whereas competitive one‐electron reduction occurs at a much lower efficiency generating FapyGua in a smaller relative yield ranging from 5.7 to 10.1% in isolated DNA exposed to either UVA excited benzophenone MQ or riboflavin in aerated aqueous solutions [39]. Furthermore, it was found that 266‐nm biphotonic ionization of the guanine moiety of ODNs [109, 110, 111, 112] and isolated DNA [59, 113, 114] gives rise to predominant 8‐oxoGua, whose yield is increased by sevenfold in duplex structure with respect to single‐stranded ODN [91]. Evidence was also provided for the formation of 2,2,4‐triamino‐5‐(2*H*)‐oxazolone (Oz) through the deprotonation pathway [115], in a slightly lower yield than 8‐oxoGua [39]. Thus, the addition of O_2_ **·** ^−^ to G(‐H)^•^ [82] leads through a rather complicated multistep pathway to the formation of 2,5‐diamino‐4H‐imidazol‐4‐one (Iz) that is slowly converted by hydrolysis (*t*_½_ = 147 min at 37°C) into Oz [115, 116]. It is worth mentioning that both Oz and Iz are alkali‐labile lesions [117, 118] that can be detected at the nucleoside level as piperidine‐sensitive sites in defined sequence DNA fragments [40, 109, 110, 111].

#### 2.2.5 Adenine

Similar to the case of guanine, hydration of adenine radical cations (Ade^•+^) in ODNs [40] and isolated DNA [39, 113, 114] gives rise to 8‐oxoAde upon one‐electron oxidation of transient 8‐hydroxy‐7,8‐dihydroaden‐7‐yl (Figure 2). In contrast to guanine, however, 8‐oxoAde is formed in less than 10% of 8‐oxoGua. Competitive one‐electron reduction of the 7‐yl radical generates 2,6‐diamino‐4‐formamidopyrimidine (FapyAde) [119, 120]. The *N*6‐yl radical arising from the deprotonation of Ade^•+^ in isolated dA is the precursor through hydrolytic deamination of 2′‐deoxyinosine, and as yet, a not fully characterized dimeric adduct arising from radical addition to intact dA [121]. Several *N*−6 substituted adenine nucleosides have been used to selectively generate by photolysis the strongly oxidizing *N*6‐yl radical in duplex DNA fragments [121, 122, 123]. The generation of the latter radical in 5′‐GTX sequences of ODNs, where X is the photoprecursor used to generate *N*6‐yl radicals, is able to abstract a hydrogen atom from the methyl group of adjacent thymine bases that upon formation of 5‐(uracilyl)methyl peroxyl radicals in the presence of O_2_, subsequently add to guanine on the 5′‐side, leading to tandem lesions, i.e.,8‐oxodG next to 5‐fodU [124]. The authors suggest that tandem lesions involving the oxidation of dT to other products, such as dTGly, can also form in sequences of 5′‐TTX and 5′‐TTTX. This pathway is supported by the absence of formation of piperidine‐induced breaks in sequences of ds‐ODNs in which the 5′‐flanking T is replaced by A (e.g., 5′‐AX). Interestingly, hole transfer continues past 5′‐AX and 5′‐UX nonproductive sequences to distant 5′‐TT dinucleotides likely via A bases on the opposite strand in ds‐ODNs [125]. The above results using an independent precursor of *N*6‐yl radicals support the idea that guanine is the major site of DNA damage upon one‐electron oxidation of the ODNs.

#### 2.2.6 One‐Electron Oxidation Reactions of Isolated DNA: Similarities and Differences with Respect to Hydroxyl Radical

Predominant one‐electron oxidation products of the 5 main canonical purine and pyrimidine bases/2′‐deoxyribonucleoides have been measured in isolated DNA by HPLC coupled to either electrochemical detection (essentially electroactive 8‐oxoGua and 8‐oxoAde) or EI mass spectrometry [75, 114]. Even if the initial radical events of the main degradation pathways of the nucleobase moieties initiated by one‐electron oxidants and ^•^OH differ, similarities have been established on a qualitative basis for base oxidation products. A major difference between the two processes concerns the hydration of thymine radical cations, which exclusively occurs at the C6 position, while ^•^OH attaches preferentially to the C5 position of pyrimidine, leading to 5‐hydroxy‐5,6‐dihydrothymin‐6‐yl [126].

In general, the mechanism of one‐electron oxidation and ^•^OH mediated reactions of DNA bases coalesce into a similar mixture of final stable products, and thus, the main differences are observed in the relative yields of each of the products. Thus, it is possible to discriminate the two classes of oxidizing agents based on the distribution of the final degradation products [127, 128, 129].

‐ Elevated formation of 8‐oxoGua and FapyGua with respect to pyrimidine oxidation products in duplex DNA, including Thy‐Gly, 5‐OHCyt, 5‐HmUra, and 5‐foUra [39, 114]. This contrasts with an almost similar distribution of the main ^•^OH‐mediated oxidation in isolated DNA [39]. The preponderant formation of both 8‐oxoGua and FapyGua in one‐electron oxidized DNA that is strongly favored by base π‐stacking is rationalized in terms of hole migration of initially formed purine and pyrimidine radical cations with preferential trapping at a distance by guanine bases [130]. The proposed mechanisms of tunneling, super exchange, and hopping of charge transport [131, 132, 133, 134], subject to extensive experimental and theoretical studies, have been critically reviewed in a recent survey [135].

‐ Strong base sequence effect on oxidatively generated damage to guanine distribution. It may be remembred that the sequence context has a modest effect on the distribution of ^•^OH‐mediated guanine oxidation products revealed by PAGE analysis as Fpg‐sensitive sites for 8‐oxoGua and FapyGua or alkali labile Oz [127, 128]. Fast migration of positive hole (radical cation) toward guanine via adenine was initially observed in dinucleotide monophosphates [136]. Evidence was provided by different groups that oxidized guanine hotspots were generated at 5′‐G in GG doublets and central G in GGG triplet by several one‐electron oxidants, including CO_3_ **·** ^−^ and photoexcited riboflavin and MQ [137, 138, 139, 140]. The higher susceptibility to oxidation of 5′G in these sequence contexts is due to a substantial lowering of its ionization potential, as suggested from theoretical studies [141, 142].

‐ Nucleophilic addition at C8 of guanine radical cation. In addition to hydration of Gua^•+^ that partly mimics ^•^OH‐mediated reactions on nucleobases in terms of oxidation product formation, several biologically relevant nucleophiles were shown to form specific stable cross‐links via covalent binding the imidazole moiety at C8. A first example of these relevant reactions is provided by the intra‐strand addition of thymine through nucleophilic N3 to C8 of 5′‐G in ss‐ODNs upon exposure to CO3^•−^. The low yield of generated G*‐T* adduct is favored when thymine and guanine are separated by an intervening cytosine [143, 144]. Another nucleophilic addition implicating G^•+^ in DNA duplexes is the efficient formation of inter‐strand cross‐links with opposite cytosine [145, 146]. However, the structure of the adduct(s) remains to be fully elucidated. DPCs represent a major class of oxidatively generated DNA damage that, for a long time, represented a challenging identification issue. Pioneering flash‐quenching experiments have clearly established that one‐electron oxidation of guanine is a critical step for the generation in model studies of DPCs in the presence of DNA‐bound proteins [147, 148]. Insights into the mechanism of one‐electron DPCs formation under the latter conditions were gained from the isolation of a stable guanine‐lysine crosslink formed in high yields by UVA‐excited riboflavin‐mediated type I photosensitized oxidation of d(TGT) in the presence of KKK peptide [149]. This was rationalized in terms of covalent attachment of the free €‐amino group of DNA‐bound trilysine peptide to C8 of Gua, followed by one‐electron oxidation of the transient generated N7‐yl radical. Similar types of cross‐links were formed upon riboflavin photosensitization of aerated aqueous solutions of ds‐DNA in the presence of short polyamines, including putrescine, spermine, and spermidine [150] known to promote stabilization and condensation of nuclear DNA [151, 152]. Interestingly, crosslink formation that involves the free amino groups of polyamines is more efficient than the competitive nucleophilic hydration of G^•+^ that leads to 8‐oxoGua. Further details on the mechanism of formation of DPCs have been provided by extensive molecular dynamic investigations [153, 154]. The high‐intensity UVC ns laser photolysis approach developed for generating DPCs is highly suitable for mapping binding sites at the nucleotide resolution between genomic DNA and structural proteins. A major advantage of the technique that has received numerous relevant applications is the real‐time dynamic visualization of labile contacts under “single pulse” conditions (for recent reviews see Angelov et al. [155] and Lone et al. [156]).

‐ Lack of direct DNA strand break formation upon the one‐electron oxidation of nucleobases. Most one‐electron oxidants do not induce SSBs. The direct effect of ionizing radiation on cellular DNA involves initial ionization of both the 2‐deoxyribose and nucleobase moieties. In turn, though, a large proportion, but not all, of 2‐deoxyribose radical cations transfer to nucleobases. The formation of a strand break by the direct effect may take place by ionization of the sugar‐phosphate backbone. In contrast, ^•^OH induces SSBs much more efficiently than direct ionization as a result of hydrogen atom abstraction at the C3, C4, and C5 of the 2‐deoxyribose moiety [15, 16].

## 3 Bi‐Photonic Ionization of Cellular DNA by 266‐nm Nanosecond Laser Irradiation

Accurate and quantitative determination of the main oxidatively generated base damage in human monocytes by high intensity UVC ns‐laser pulses was assessed by isotopic dilution HPLC‐ESI‐MS/MS analysis operating in the multiple reacting monitoring mode [114, 157, 158]. 8‐OxodG is by far the predominant DNA oxidation modification over minor amounts of dT‐Gly, 5‐HmdU, 5‐fodU, suppressed and 8‐oxodA (Table 1). Similarly, the yield of 8‐oxodG is overwhelming in comparison to other products, i.e., 5‐OHdC and ultra‐minor T*‐G* intra‐strand adduct yields as measured in the DNA of HeLa cells following exposure to 266‐nm laser radiation [160]. The distribution profile of the oxidized bases is indeed similar to the modification pattern obtained by bi‐photonic one‐electron oxidation of isolated DNA [114]. This provides strong support for the occurrence of efficient electron transfer to the nucleobases with the lowest oxidation potential in cellular DNA, which is guanine [23, 24].

**TABLE 1:** Formation of oxidatively generated base damage in cellular DNA by high intensity UVC nanosecond laser pulses (50 mJ.cm^2^) and ionizing radiation: number of modifications per 10^9^ nucleosides either per one mJ laser energy or per Gy. Most modifications are detected as the corresponding 2’‐deoxyribonucleoside (for structures see Figures 1 and 2).

Lesions	Gamma rays^a^	UVC laser^b^
dT‐Gly	97	160
5‐HmdU	29	60
5‐fodU	22	20
8‐oxodG	20	1.290
FapyGua	39	c
8‐oxodA	3	c
FapyAde	5	c

a From Pouget et al. [159].

b From Douki et al. [157].

c not determined.

As a striking difference, a pronounced change was noted in the distribution of oxidatively generated base modifications upon exposure of cellular DNA to ionizing radiation [157, 159, 161, 162]. Under these irradiation conditions, 8‐oxoGua is formed with a lower abundance than dT‐Gly, 5‐HmdU, and 5‐fodU. Thus, the contribution of the direct ionization of DNA appears to be minor with respect to the predominant implication of indirectly generated ^•^OH to radiation‐induced damage to cellular DNA [157, 163, 164]. This is in agreement with previous estimations that have proposed that the latter indirect effect is responsible for two‐thirds of overall genome degradation [165, 166].

It has recently been reported that the presence of bicarbonate in gamma‐irradiated cells is expected to significantly modify the ^•^OH‐mediated oxidation reactions of DNA [167]. This was explained by the conversion of ^•^OH into CO_3_ **·** ^−^ in the presence of physiological concentrations of bicarbonate, such that the ensuing CO_3_ **·** ^−^ diverts the pathway of DNA damage from predominantly ^•^OH reactions with DNA base and 2‐deoxyribose moieties, to selective one‐electron oxidation of guanine. Thus, the proposed scenario in which ^•^OH is converting into CO_3_ **·** ^−^ should result in the overwhelming formation of oxidatively generated guanine degradation products and the reduced formation of DNA strand cleavage. Furthermore, it was also shown that guanine modifications and oxidized pyrimidine ring degradation products revealed as Fpg‐ and endo III‐ sensitive sites, respectively, were formed in similar amounts together with a twofold higher yield of direct DNA strand breaks/alkali‐labile sites [168, 169]. The results of the above experiments suggest that CO_3_ **·** ^−^ does not significantly contribute to radiation‐induced damage to cellular DNA [164].

## 4 Conclusions and Perspectives

The use of high‐intensity 266‐nm ns‐laser radiation has been found to be a suitable tool to selectively ionize the four purine and pyrimidine bases of cellular DNA according to a bi‐photonic process. This has allowed, in association with accurate and quantitative ESI‐tandem mass spectrometry measurements, to decipher the oxidative degradation pathways following the decay of the base radical actions. The specific distribution profile of oxidized nucleobases characterized by the preeminence of guanine degradation products can be used as a molecular signature of the mode of action of one‐electron oxidants. Forthcoming studies should be aimed at characterizing in cellular DNA other specific nucleophilic adducts to guanine radical cations, including DNA interstrands (ICLs) and DPCs.

## Funding

FAPESP, Fundação de Amparo à Pesquisa do Estado de São Paulo; Grants 2024/16848−3, 2026/00278−9, 2026/00280−3, 2022/26871−5, 2022/11371−9; FAPESP/INCT 202526871−5, and CEPID Redoxoma 2013/07937−8, CNPq (Conselho Nacional de Desenvolvimento Científico e Tecnológico; Grants 304350/2023−0, 304945/2021−8)), INCT, Instituto Nacional de Ciência e Tecnologia em Metodologias Quantitativas e de Precisão em Biomedicina Redox, grants 408213/2024−8. PRPUSP (Pro‐Reitoria de Pesquisa da Universidade de São Paulo; NAP Redoxoma 2011.1.9352.1.8). This work was supported by grants from the Natural Sciences and Engineering Research Council of Canada (NSERC, grant number RGPIN‐2022−03974) and the Canadian Institutes of Health Research (CIHR, grant number PJT‐195674).

## Conflicts of Interest

The authors declare no conflicts of interest.

## Data Availability

The data that support the findings of this study are available on request from the corresponding author. The data are not publicly available due to privacy or ethical restrictions.
